# Antimicrobial usage in cattle and poultry production in Dar es Salaam, Tanzania: pattern and quantity

**DOI:** 10.1186/s12917-021-03056-9

**Published:** 2022-01-03

**Authors:** Rogers Azabo, Stephen Mshana, Mecky Matee, Sharadhuli I. Kimera

**Affiliations:** 1 Department of Veterinary Microbiology, Parasitology and Biotechnology, College of Veterinary Medicine and Biomedical Sciences, Sokoine University of Agriculture, Morogoro, Tanzania; 2 National Livestock Resources Research Institute, Kampala, Uganda; 3 SACIDS Foundation for One Health Sokoine University of Agriculture, Morogoro, Tanzania; 4 Department of Microbiology/Immunology, Weill Bugando School of Medicine, Catholic University of Health and Allied Sciences, Bugando, Mwanza, Tanzania; 5 Department of Microbiology and Immunology, School of Medicine, Muhimbili University of Health and Allied Sciences, Dar es Salaam, Tanzania; 6 Department of Veterinary Medicine and Public Health, College of Veterinary Medicine and Biomedical Sciences, Sokoine University of Agriculture, Morogoro, Tanzania

**Keywords:** Antimicrobial use, Cattle, Dar Es Salaam, Poultry, Practices, Quantity, Tanzania

## Abstract

**Background:**

Antimicrobials are extensively used in cattle and poultry production in Tanzania. However, there is dearth of information on its quantitative use. A questionnaire-based cross-sectional study was conducted from August to September 2019 in randomly selected poultry and small-scale dairy farms, in three districts of Dar es Salaam City eastern, Tanzania, to assess the practice and quantify antimicrobial use. Descriptive and statistical analyses were performed at a confidence interval of 95%. The ratio of Used Daily Dose (UDD) and Defined Daily Dose (DDD) were used to determine whether the antimicrobial was overdosed or under dosed.

**Results:**

A total of 51 poultry and 65 small-scale dairy farms were involved in the study. The route of antimicrobial administration was 98% orally via drinking water and 2% in feeds for poultry and for small-scale dairy farms, all through parenteral route. Seventeen types of antimicrobials comprising seven classes were recorded in poultry farms while nine belonging to six classes in the small dairy farms. Majority of the farms (poultry, 87.7% and small scale dairy, 84.3%) used antimicrobials for therapeutic purposes. About 41% of the poultry and one third (34%) of the dairy farmers’ were not compliant to the drug withdrawal periods. Beta-lactams, fluoroquinolones, sulphonamides, tetracyclines and macrolides were the commonly used antimicrobials on these farms. In the poultry farms both those with records and those which relied on recall, antimicrobials were overdosed whereas in the small dairy farms, sulfadimidine, oxytetracycline and neomycin were within the appropriate dosing range (0.8–1.2). The majority (58.6%) of farmers had adequate level of practices (favorable) regarding antimicrobial use in cattle and poultry production. This was associated with the age and level of education of the cattle and poultry farmers.

**Conclusion:**

The study revealed a widespread misuse of antimicrobials of different types and classes in both poultry and small-scale dairy farming in Dar es Salaam, Tanzania. This result gives insight into the antimicrobial use practices and its quantification. The information obtained can guide and promote prudent use of antimicrobials among the farmers by developing mitigate strategies that reduce antimicrobial resistance risk potentials.

**Supplementary Information:**

The online version contains supplementary material available at 10.1186/s12917-021-03056-9.

## Background

Antimicrobial use in food animals has become an issue of global concern [1] due to its association with emergence of antimicrobial resistance (AMR) pathogens [2]. In Tanzania like any other low-middle income country, antimicrobial usage in animal production is weakly regulated and restricted [3].This has contributed to injudicious use of antimicrobials leading to the emergence and spread of resistance [4, 5]. There is also evidence that lack of basic knowledge on the concept of AMR among livestock keepers may exacerbate the problem [6].

The increased antimicrobial usage in agriculture is driven by intensification in livestock production and demands for animal products [7]. Several studies have indicated indiscriminate use of antimicrobials in these livestock species [3, 8–10], fuelled by access without veterinarian prescription and expansion of the antimicrobial drug trade [6] to include non-therapeutic purposes [11]. The most commonly used antimicrobials in livestock production among the rural population in northern Tanzania are tetracyclines, pencillins, aminoglycosides, macrolides, and sulphonamides [2].

Despite the numerous studies on antimicrobial use in livestock [2–10], there is dearth of information on its practice and quantification in poultry and dairy cattle production in Tanzania especially in peri-urban / urban population Therefore, understanding the Antimicrobial use (AMU) practices of producers and precisely quantifying exposure to antimicrobials is critical for the success of interventions to improve AMU in cattle and poultry production.

The aim of this study is to assess farmers’ antimicrobial use practices and quantification of antimicrobials used in poultry and small-scale dairy farms in Dar es Salaam, Tanzania. The findings of this study are likely to increase awareness of both “irrational” and “rational” uses of antimicrobials in food producing animals and also provide evidence based information to policy makers.

## Results

### Farmers’ socio-demographic characteristics

**Fig. 1:**
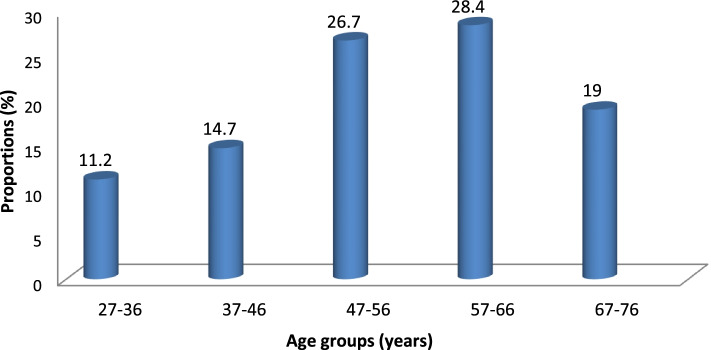
Proportion of age distribution of cattle and poultry farmers in Dar-es-Salaam, Tanzania

The respondents in this study had a mean age of 54.5 ± 12.1 SD years and a majority (28.4%) were in the age group (57–66) years (Fig.1). Most of the respondents interviewed were male (60%), especially in the dairy farmer group (65%). With regard to education (35.3%) had achieved tertiary education level, 29.3% had secondary education, (33%) primary education and 3(2.6%) not educated.

About 71% of the farmers indicated that livestock production was their main occupation. In addition, 62% of farmers had over 6 years of livestock rearing or management experience (Table 1).

**Table 1:** Farmers’ socio-demographic characteristics in Dar es Salaam, Tanzania

Variable	Number of farmer categories, n (%)			
	Cattle (65)	Poultry (51)	Overall (116)	Confidence interval (95%)
**Gender**				
M	42(64.6)	27(52.9)	69(59.5)	50.4,67.9
F	23(35.4)	24(47.1)	47(40.5)	32.0,49.6
**Age (years)**				
27–36	2(3.1)	11(21.6)	13(11.2)	6.7,18.2
37–46	10(15.4)	7(13.7)	17(14.7)	9.4,22.2
47–56	17(26.2)	14(27.4)	31(26.7)	19.5,35.4
57–66	19 (29.2)	14(27.4)	33(28.4)	21.0,37.3
67–76	17(26.2)	5(9.8)	22(19.0)	12.9,27.1
**Education**				
Informal	1(1.5)	2(3.9)	3(2.6)	0.9,7.3
Primary	21(32.3)	17(33.3)	38(32.8)	24.9,41.7
Secondary	19(29.2)	15(29.4)	34(29.3)	21.8,38.2
Tertiary	24(36.9)	17(33.3)	41(35.3)	27.2,44.4
**Main occupation of respondent**				
Livestock	44(67.7)	38(74.5)	82(70.7)	61.9,78.2
Others	21(32.3)	13(25.5)	34(29.3)	21.8,38.2
**Experience in livestock rearing (years)**				
≤ 6	31(47.7)	13(25.5)	44(37.9)	29.6,47.0
> 6	34(52.3)	38(74.5)	72(62.1))	52.9,70.4

### Practices of antimicrobial usage in cattle and poultry

In this study, 23 (19.8%) of the farms visited had antimicrobial use records while 93 (80.2) relied on recall (Additional file 1, Tables 1&3). In poultry production, 14 (27.5%) of the farms had antimicrobial use records while 37(72.5%) farms relied on recall. Meanwhile in cattle production, 9 (13.8%) of the farms had antimicrobial use records while 56(86.2%) relied on recall. However, in both scenarios the farmers had empty and newly used antimicrobial bottles and sachets. In cases where information was not sufficient online summary characteristic of the product was accessed.

There was no significant difference between the two antimicrobial data collection methods (records vs recall) and the Defined Daily Dose (DDD) at a probability cut-off of 0.05.

### Qualitative antimicrobial use estimate

Of the 116 farms visited 65 (56%) were small-scale dairy farms while 51(44%) poultry farms and all of them used antimicrobials. On personnel that provided farmers with information on drug source, 82.8% (95%CI, 74.9, 88.6) mentioned veterinarians, including 83.1% of the dairy and 82.4% of poultry farmers while 3.4% reported neighbours of whom 3.1% were dairy cattle farmers and 3.9% poultry farmers. Across all the farmer categories, the primary purpose of antimicrobial use was for treatment 83.6% (95%CI, 75.8, 89.3) followed by “treatment and prophylaxis” 13.8% (95%CI, 8.6, 21.2), prophylaxis 1.7% (95%CI, 0.5, 6.1) and growth promotion 0.9% (95%CI, 0.2, 4.7). About 89.2% of the small-scale dairy farmers indicated that treatment was their primary purpose for antimicrobial, compared with 76.5% of the poultry farmers.

When asked where they purchased antimicrobials used on cattle and poultry, 44% (95%CI, 35.3, 53.1) of the farmers said veterinary drug shops, 16.4% (95%CI, 10.7, 24.2) veterinary clinics while 39.7% patronized individual veterinarians (95%CI, 31.2, 48.8). Notable, 43.1% of the dairy cattle farmers indicated veterinary drug shops while 45.1%of the poultry farmers indicated so. 90.5% (95%CI, 83.8, 94.6) of the farms signaled that they engaged services of veterinarians/animal health, especially the dairy cattle farmers (90.8%) while 9.5% (95%CI 5.4, 16.2) of the farmers practiced self- administration.

In all the dairy farms visited, antimicrobials were administered through parenteral route while in 98% of the poultry farm orally via drinking water and 2% in feeds. Majority (65.7%) were non-compliant to withdrawal periods, of whom, 63.1% were dairy cattle farmers and 66.7% were poultry farmers. Most farmers, 66.4% (95%CI, 57.4, 74.3) stored their antimicrobials in cupboards, followed by open shelf indoors, 24.1% (95%CI, 17.3, 32.7) and shelf direct sunlight, 9.5% (95%CI 6.4, 16.2), with some variations in the different farmer groups (Table 2).

**Table 2:** Practices of antimicrobial usage in livestock production by Farmers in Dar es Salaam, Tanzania

Practice	Producer categories, n (%)			
	Cattle (65)	Poultry (51)	Overall (116)	Confidence interval (95%)
**Source of drug information to the farmer**				
Veterinarian	54(83.1)	42(82.4)	96(82.8)	74.9,88.6
Household experience	9(13.8)	7(13.7)	16(13.8)	8.7,21.2
Neighbours	2(3.1)	2(3.9)	4(3.5)	1.4,8.5
**Purchasing place for antimicrobials**				
Veterinary drug shops	28(43.1)	23(45.1)	51(44.0)	35.3,53.1
Veterinary clinic	13(20.0)	6(11.8)	19(16.4)	10.7,24.2
Individual veterinarian	24(36.9)	22(43.1)	46(39.7)	31.2,48.8
**Purpose for antimicrobial usage**				
Therapeutic	58(89.2)	39(76.5)	97(83.6)	75.8, 89.3
Prophylaxis	1(1.5)	1(1.9)	2(1.7)	0.5,6.1
Therapeutic & Prophylaxis	6(9.2)	10(19.6)	16(13.8)	8.7,21.2
Growth promotion	0(0.0)	1(1.9)	1(0.9)	0.2,4.7
**Drug sellers asking for prescriptions**				
Yes	3(4.6)	2(3.9)	5(4.3)	1.9,9.7
No	58(89.2)	45(88.2)	103(88.8)	81.8,93.3
Sometimes	4(6.2)	4(7.8)	8(6.9)	3.5,13.0
**Administration of drug to livestock**				
Veterinarian/Animal health worker	59(90.8)	46(90.2)	105(90.5)	83.8,94.6
Self	6(9.2)	5(9.8)	11(9.5)	5.4,16.2
**Route of antimicrobial administration**				
Parenteral (Injection)	65(100)	0(0.0)	65(56.0)	46.9,64.7
Water	0(0.0)	50(98.0)	50(43.1)	34.5,52.2
Feeds	0(0.0)	1(2.0)	1(0.9)	0.2, 4.7
**Compliance with drug withdrawal period**				
Yes	24(36.9)	17(33.3)	41(35.3)	27.2,44.4
No	41(63.1)	34(66.7)	75(64.7)	55.6,72.8
**Antimicrobial storage**				
Cupboard	43(66.2)	34(66.7)	77	57.4,74.3
Open shelf indoor	16(24.6)	12(23.5)	28	17.3,32.7
Shelf direct sunlight	6(9.2)	5(9.8)	11	5.4,16.2

Overall, 58.6% of the livestock farmers had adequate level of practices (favorable) in accordance to good antimicrobial use based on their responses. Farmers in the age group 27–36 years were four times more likely to have unfavorable antimicrobial use practices than those in the age group 67–76 years (OR = 3.88; 95% CI = 1.71–6.05; *p* = 0.001). Meanwhile, farmers with low educational qualifications (Primary school qualifications and below) were three times more likely to have unfavorable antimicrobial use practices than those with tertiary education (OR = 2.71; 95% CI = 1.44–3.98; *p* = 0.001) (Table 3).

**Table 3:** Socio-demographic characteristics of cattle and poultry farmers associated with antimicrobial use practices in Dar es Salaam

Variable	Number of respondents n (%)	Unfavorable practices, n (row %)	Odds ratio (OR)	95%Confidence Interval	P-value
**Age(in years)**					
27–36	13(11.2)	10 (76.9)	3.88	1.71,6.05	0.001
37–46	17(14.7)	11 (64.7)	2.60	0.85,4.36	0.004
47–56	31(26.7)	13 (41.9)	1.19	−0.33,2.73	0.124
57–66	33(28.4)	10 (30.3)	0.33	−1.23,1.89	0.678
67–76	22(19.0)	4 (18.2)	1.00		
**Farmers’ level of Education (Educ2)**					
Primary	41(35.3)	10 (24.4)	1.00		
Secondary	34(29.3)	9 (26.5)	0.23	−1.03,1.49	0.716
Tertiary	41(35.3)	29 (70.7)	2.71	1.44,3.98	0.001

Statistically significant at *p* < 0.05

### Antimicrobials frequently used in poultry and cattle production

**Fig. 2:**
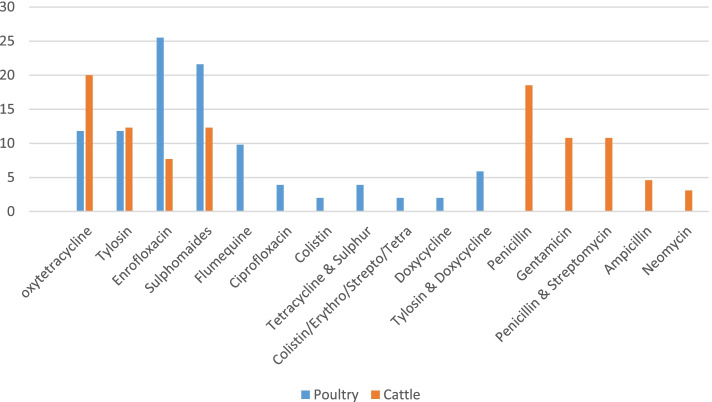
Antimicrobials commonly used by poultry and cattle farmers in Dar-es-Salaam, Tanzania

A total of 17 antimicrobials were used among the poultry farms, which comprised of 7 classes (Additional file 2, Table 1&2). The most frequently used antimicrobials in poultry production were: enrofloxacin 25.5%), followed by sulphonamides (21.6%), oxytetracycline (11.8%), tylosin (11.8%) 195 and flumequine (9.8) (Fig. 2).

In the small-scale dairy farms, 13 different types of antimicrobials comprising 7 classes were used (Additional file 2, Table 3 & 4). The frequently used antimicrobials were oxytetracycline (20.0%), followed by penicillin (18.5%), sulphonamides (12.3%), tylosin (12.3%), penstrep (10.8%), gentamicin (10.8%), enrofloxacin, 5(7.7%); ampicillin (4.6%) and neomycin (3.1%) (Fig. 2).

### Quantitative antimicrobial use

This is different from the former, in that it deals with the amount of antimicrobials used. The DDD and UDD in both the poultry and cattle farms were determined based on the information in Additional file 1, (Table 2 & 4) and also statistical data summary on the antimicrobials recalled, Table 5 in the same file. In the poultry farms with antimicrobial use records, sulfamethoxypyridazine (46.1%) was the most frequently used antimicrobial agent, followed by oxytetracycline (19%), tylosin (14.2%) and enrofloxacin (11%) while those farms which relied on recall, sulfamethoxypyridazine (28.8%) was still the most frequently used antimicrobial agent, followed by oxytetracycline (18.5%), tylosin (13.9%) and enrofloxacin (9.6%) (Table 4). In the small-scale dairy farms with antimicrobial use records, penicillin (36.4%) was the most frequently used antimicrobial agent, followed by sulfamethoxazole (22.3%), oxytetracycline (14.3%) and dihydrostreptomycin (11.5%) while those which relied on recall, pencillin (43.1%) still was the most frequently used antimicrobial agent, followed by dihydrostreptomycin (16.4%), sulfamethoxazole (8.9%) and gentamicin (6.8%) (Table 5).

**Table 4:** Daily dosages (mg/kg), dosing ratios and total amount of antimicrobials used (g) in surveyed poultry farms with records and those that relied on recall in Dar es Salaam, Tanzania

Antimicrobial class	Antimicrobial name	DDD	UDD	UDD/DDD	Total used [g (%)]
**Antimicrobials from farms with records**					
Diaminopyrimidines	Trimethoprim	5.0	18.6	3.7	774.6 (9.0)
Fluoroquinolones	Enrofloxacin	20.3	60.8	2.9	919.4 (11.0)
Macrolides	Tylosin	9.5	32.4	3.4	1184.7 (14.2)
Polymyxins	Colistin	5.3	15.8	2.9	23.7 (0.3)
Sulphonamides	Sulfadiazine	11.3	33.8	2.9	33.8 (0.4)
	Sulfamethoxypyridazine	28.1	112.5	4.0	3839.2 (46.1)
Tetracyclines	Oxytetracycline	45.0	135.0	3.0	1552.6 (19.0)
**Antimicrobials from farms which relied on recall**					
Aminoglycosides	Streptomycin	7.9	23.6	2.9	9.5 (0.4)
Diaminopyrimidines	Trimethoprim	8.8	26.4	3.0	149.2 (6.4)
Fluoroquinolones	Ciprofloxacin	13.5	40.5	3.0	32.4 (1.4)
	Enrofloxacin	18.3	56.2	3.1	223.5 (9.6)
	Flumequine	21.0	63.0	3.0	156.6 (6.7)
Macrolides	Erythromycin	7.9	23.6	2.9	9.5 (0.4)
	Tylosin	14.1	43.3	3.1	324.9 (13.9)
Polymyxins	Colistin	2.5	7.4	2.9	3.0 (0.1)
Sulphonamides	Sulfadiazine	26.7	80.1	3.0	95.9 (4.1)
	Sulfadimerazine	22.5	67.5	3.0	13.5 (0.6)
	Sulfadimidine	33.3	99.9	3.0	137.8 (5.9)
	Sulfamethoxypyridazine	42.2	127.1	3.0	671.6 (28.8)
Tetracycline	Oxytetracycline	26.5	79.6	3.0	431.9 (18.5)
	Doxycycline	18.6	55.7	2.9	71.6 (3.1)

Defined Daily Dose (DDD), Used Daily Dose (UDD), Total used = Total volumes of antimicrobial used (grams)

**Table 5:** Daily dosages (mg/kg), dosing ratios and total amount of antimicrobials used (g) in surveyed small scale dairy farms with records and those that relied on recall in Dar es Salaam, Tanzania

Antimicrobial class	Antimicrobial name	DDD(mg/kg)	UDD(mg/kg)	UDD/DDD	Total used [g (%)]
	**Antimicrobials from farms with records**				
Beta-lactam	Penicillin	16.0	47.5	2.9	404.0 (36.4)
Aminoglycosides	Dihydrostreptomycin	10.0	30.0	3.0	127.5 (11.5)
	Gentamicin	5.0	14.8	2.9	44.1 (4.0)
Diaminopyrimidines	Trimethoprim	3.4	7.5	2.2	44.9 (4.0)
Fluoroquinolones	Enrofloxacin	7.5	21.9	2.9	83.7(7.5)
Sulphonamides	Sulfamethoxazole	17.0	37.4	2.2	247.2 (22.3)
Tetracycline	Oxytetracycline	10.0	15.8	1.6	159.0 (14.3)
	**Antimicrobials from farms which relied on recall**				
Beta-lactam	Ampicillin	11.1	30.6	2.8	174.0 (4.2)
	Penicillin	16.0	34.3	2.1	1791.1(43.1)
Aminoglycosides	Dihydrostreptomycin	11.1	36.2	3.3	679.0 (16.4)
	Gentamicin	5.0	14.2	2.8	282.0 (6.8)
	Neomycin	20.0	24.7	1.2	150.0 (3.6)
Diaminopyrimidines	Trimethoprim	2.6	5.4	2.1	91.7 (2.2)
Fluoroquinolones	Enrofloxacin	5.6	10.6	1.8	235.2 (5.7)
Sulphonamides	Sulfadiazine	12.5	35.3	2.8	90.0 (2.2)
	Sulfadimidine	33.3	28.2	0.8	59.9 (1.4)
	Sulfamethoxazole	13.1	25.1	1.9	368.4 (8.9)
Tetracycline	Oxytetracycline	10.0	9.6	0.9	230.3 (5.5)

Defined Daily Dose (DDD), Used Daily Dose (UDD), Total used = Total volumes 223 of antimicrobial used (grams)

The averagely applied dosages to poultry and small-scale dairy farms, described as Defined Daily Dose (DDD) and Used Daily Dose (UDD) are presented above in Tables 4 and 5, respectively. From the ratio UDD to DDD it can be seen that in the poultry farms irrespective of antimicrobial use records or recall, all antimicrobials were over dosed, the same to the small-scale dairy farms with records. For those farms which relied on recall of antimicrobials in the small scale dairy farms, the ratio of UDD to DDD indicated that sulfadimidine, oxytetracycline and neomycin were appropriately dosed within the dosing range (0.8–1.2) while the rest of antimicrobials were overdosed refer to Table 5.

## Discussion

This study revealed that antimicrobial use was a common practice among the poultry and cattle farmers and its use was 100% in both enterprises. Most of the antimicrobials were for treatment purposes and were mainly obtained from the veterinary drug shops. Both poultry and cattle farmers obtained their antimicrobial use information from the veterinarians. We found out that most farmers were not compliant with drug withdrawal periods. A variety of antimicrobials were used in the farms in the study area and the commonly used were tetracyclines, sulphonamides, fluoroquinolones macrolides, penicillin, penstrep and gentamicin. The study also found out that most of the antimicrobials were overdosed irrespective of the farmers having records or relied on recall while sulfadimidine, oxtetracycline and neomycin, were within the dosing range in cattle farms for those that relied on recall.

In poultry, in addition to therapeutic and /or prophylactic purposes, antimicrobial agents were used as growth promoters to a less extent. This finding is consistent with an earlier work done in Tanzania, [10] and elsewhere in Africa [12–14] but at variance with a study done in Ibadan, Nigeria by Olatoye [15] who reported that 86% of the poultry farms used antimicrobials for growth promotion. The small-scale nature of the investigated farms could probably hinder farmers from being financially buoyant to afford antimicrobials to be added to chicken feeds as growth promoters’ continuously.

Regarding small-scale dairy farms, a high antimicrobial use was either for therapeutic and/ or prophylactic purposes or both. This is in agreement with the findings by other workers [3, 16, 17]. These uses have improved animal health, sustained productivity and reduced food borne pathogens [18]. However, regular use of these antimicrobial agents by the small-scale dairy farmers may result in the diminishing efficacy of the drugs due to development of bacterial resistance, [19]. It is worth noting that, the results in this study showed that the majority of the farmers used antimicrobials for therapeutic purposes. This concurs with the study done in Peru [20].

Concerning our observation on antimicrobial usage among the farmers we visited in Dar es Salaam, although most of them are educated, a larger percentage were small scale farmers and thus in addition to sustenance of production, dependence on antimicrobials was probably due to lack of biosecurity, unhygienic practices and poor environmental sanitation which use may facilitate the emergence and spread of antibiotic-resistant pathogens [21].

We observed variations in the choice of antimicrobial agents used on the poultry and small-scale dairy farms in the study areas. This probably reflects the numerous numbers of manufacturers involved in antimicrobial production for veterinary use and thus farmers have opportunities to make choices on antimicrobial agents at will. In poultry, in both categories (those with records and those who relied on recall) fluoroquinolones, tetracyclines, sulphonamides and macrolides while in cattle (those with records and recall), tetracyclines, penicillin, sulphonamides and macrolides were the most dominantly used classes of antimicrobials. This concurs with previous studies in Tanzania [3, 6, 10] and elsewhere in Africa [22, 23] which revealed that these antimicrobials are commonly used in livestock production.

Quantitatively in poultry, the most commonly used antimicrobial class was sulphonamides followed by, tetracyclines, macrolides and fluoroquinolones. This is in agreement with the work done in Cameroon by [21]. While in the dairy, Beta-lactam/penicillin, sulphonamides, aminoglycosides and tetracycline were the most dominantly used classes of antimicrobials. This was in agreement with the work done in Nigeria and Peru [20, 23, 24] whereby the frequently used antimicrobial classes were tetracycline (oxytetracycline), followed by beta-lactam/aminoglycoside (penicillin with or without streptomycin) and trimethoprim-sulphonamides. Similarly, a previous study by [19] in Pennsylvania reported that tetracycline and penicillin were the most frequently used antibiotics in dairy cattle. A study by [6] in Kinondoni district, Tanzania also revealed that tetracycline and sulfadimidine were mostly used. In the dairy quantitatively, the most commonly used antimicrobials were sulphonamides followed by beta-lactamase, aminoglycosides, tetracyclines and macrolides. These antimicrobial agents are frequently used by poultry and dairy producers probably because they are easily acquired across the counter, relatively inexpensive, and cost-effective as when contrasted to third generation antimicrobial agents. Besides, almost all antimicrobials were overdosed in both poultry and dairy farms apart from sulfadimidine, oxytetracycline and neomycin which were within the dosing range (0.8–1.2) in small scale dairy farms. However, it should be noted that underdose of antimicrobial use has been linked to antimicrobial resistance emergence in food producing animals [25] as reported in Kenya by [26] in chicken meat.

Most of the antimicrobials encountered in the poultry and dairy farms visited in this study were either classified as critically or highly important for humans [27].The antimicrobial formulation classified under critically important are (fluoroquinolones) or highly important (sulphonamides, tetracyclines). The use of fluoroquinolones in food producing animals is worrisome as it is effective in the treatment of human enteric infection. It has been reported that in farm animals where it is used, it is associated with increased resistance in human exposed to it [28]. Fluoroquinolone has gained increased use due to its broad-spectrum activity, oral preparation and potency and lack of restrictions among other factors. The use of banned substances such as colistin in China as a growth promoter is also of concern [29]. Colistin is very valuable to treat nosocomial infections caused by multidrug-resistant gram-negative bacteria in humans [30].

As observed in the previous poultry studies in Sudan [31] and Nigeria [14] the present study showed that most farmers administered antimicrobials via drinking water and only one in feeds. In this regard whichever way is used [32] there is imprecise dosing whereby an animal can decide on the amount of either water or feed to be consumed which has effect on potentially increasing selection for resistance. In the dairy, the antimicrobials were administered through parenteral route. This is in agreement with studies done elsewhere in Africa [23]. This is probably due to the fact that most formulations are prepared to be applied through the parenteral routes.

Although the results in the present study indicate that the majority of poultry and dairy farmers complied with the services of qualified personnel for drug prescription, antimicrobial use in Tanzania is still a problem. In poultry in the study done elsewhere in Africa [12, 22] antimicrobial prescription was done by qualified personnel. We found out that a quarter of the farms practiced self-medication and a third did not comply with the antimicrobial withdrawal periods. This concurs with what [22, 33] reported in studies in Cameroon that a small proportion of poultry farms surveyed did not rely on prescriptions by veterinarians and were also not compliant with antimicrobial withdrawal periods. Similarly in the dairy, a non-negligible proportion of farms investigated did not rely on prescription by veterinarians and did not comply with withdrawal periods. This is consistent with the previous work done by [34] in Nigeria but contrary to the results reported in Boston by [35] where in addition to reliance on veterinary antimicrobial use recommendations, dosage and withdrawal periods were observed among cattle farmers. All in all, both in poultry and dairy farming self-administration may be linked to improper antimicrobial use and noncompliance to withdrawal durations to high concentrations of antimicrobials in animal products. In Tanzania this could be attributed to lack of enforcement of legislations concerning antimicrobial application to farm animals in Tanzania [10].

This study has revealed that the majority of respondents had adequate level of antimicrobial usage practices in poultry and cattle production. This may be explained by the fact that these farmers are located within the urban areas where they have access to veterinary guidance and advisory services.

We found socio-demographic characteristics of age and education to have influence on unfavorable practices of antimicrobial usage. This is likely to result into misuse of antimicrobials and hence emergence of antimicrobial resistance. Health education of the farmers through mass media like radio is imperative for behavior modification and social change towards proper antimicrobial usage practices [24].

Understanding antimicrobial use pattern currently in cattle and poultry production is of importance in developing mitigation options for judicious antimicrobial usage, which may potentially reduce antimicrobial resistance (AMR) risks in animal production [36].

In general, practices related to non-prudent antimicrobial usage were noted in this study and this may imply increased emergence of antimicrobial-resistant bacteria from farm animals to humans through various pathways. Therefore trainings and awareness increase campaigns on antimicrobials, their usage and resistance should be promoted among the livestock officers/ veterinarians. This is because we documented non-prudent AMU practices and secondly veterinarians/livestock officers are involved in antimicrobial administration. We also noted that those farmers who self-administered drugs did not have antimicrobial use records and thus need of awareness campaigns. This will help the participants to mitigate non prudent use and thus decrease the spread of resistance.

This study has some limitations. Even though the questionnaire used as data collection tool was pre-tested to ensure quality control, data was captured over a short period from respondents. This implies that the collected data can misrepresent true dispositions and recall bias especially for those farmers who are not administering their antimicrobials and also those who lack records completely.

Since most of the respondents relied on self-recall this may have affected the findings of the study. Self-recall can be affected by reporting and social desirability bias [20]. A better method of AMU data collection could be use of bins since they represent a convenient and fairly accurate ways of recording antimicrobial drug use [37] at regular intervals over a long period of time.

## Conclusion

The present study has revealed that, majority of the respondents had adequate level (58.6%) of antimicrobial use practices and few moderate level (41.4%). This could probably be associated with availability of veterinary services. Self-administration and non-compliance to antimicrobial withdrawal periods, has a public health implication as it may result in medication failure, AMR development and occurrence of drug residues in food animal products like meat, milk or eggs. Secondly the study provides a basis for development and enforcement of policies on antimicrobial use in food producing animals with a view to safeguard public health. Although it is not possible for the data in this study to be extrapolated to other parts of Tanzania, the data collection instrument can easily be used elsewhere.

## Methods

### Study design

A cross-sectional study design was used to solicit information on antimicrobial use from poultry and small scale dairy production farmers.

### Study area and target population

**Fig. 3:**
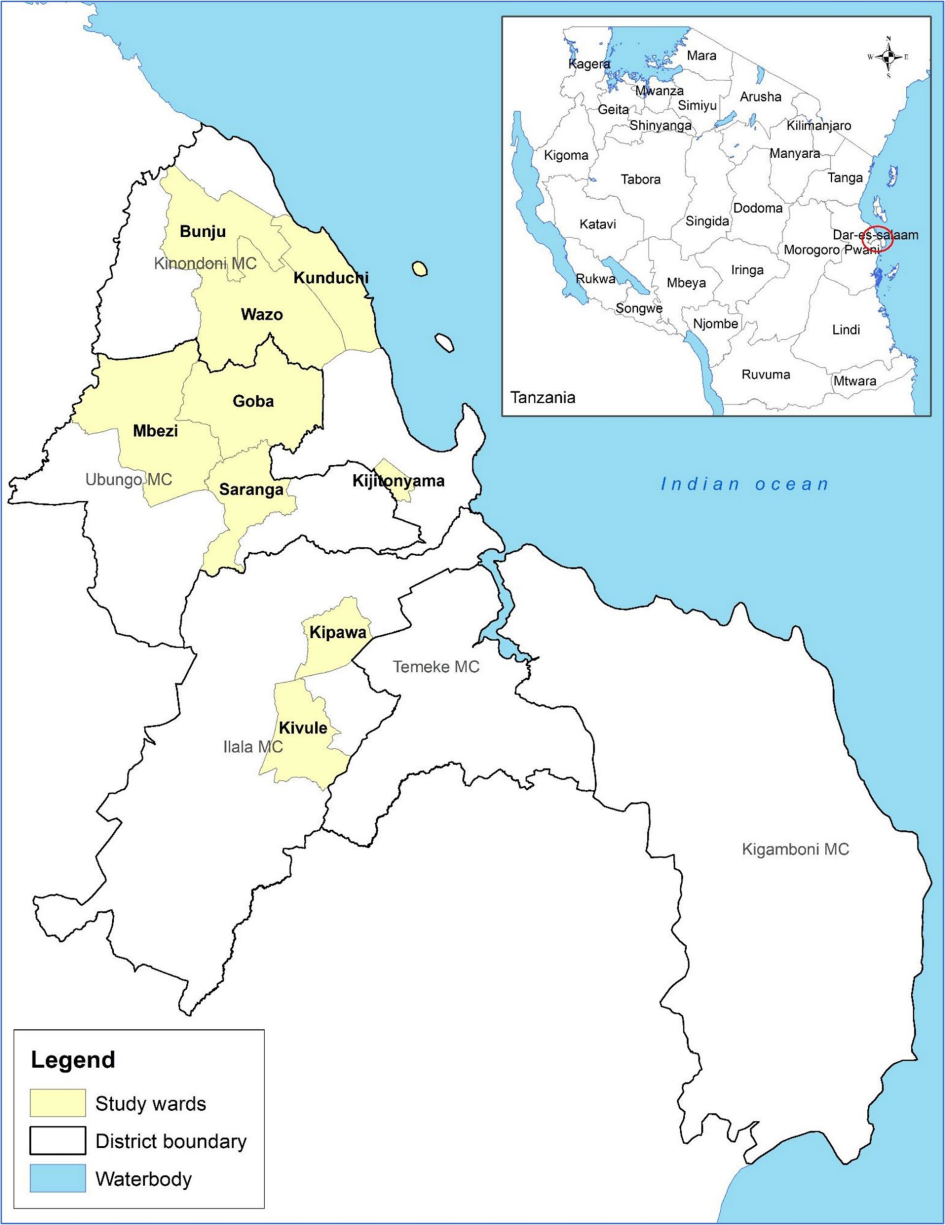
Map of the study districts (wards) in Dar- es- Salaam, Tanzania

The study was conducted from 15th August to 30th September 2019 in three districts of Dar es Salaam city, Eastern Tanzania which were purposively selected. It covered nine wards (administrative units), namely Kipawa and Kipungu in Ilala district, Bunju, Kijitonyama, Kunduchi and Wazo in Kinondoni district and Goba, Mbezi and Saranga in Ubungo district as shown in Fig. 3. These areas were selected based on their relatively high livestock activities; species of interest found and unregulated access to veterinary drugs.The target populations were farmers with poultry and small-scale dairy farms, aged 18 years and above, with a population of 100 or more birds and those with 2 or more dairy cattle.

### Farm selection

Farm selection combined random and purposive sampling based on its location. A list of 212 poultry and Small-scale dairy farms in the study districts, from which 146 farms were initially selected randomly for this survey, was provided by the District Veterinary Officers. In instances where farms were inaccessible due to distance or unwillingness of farmers to participate, they were substituted with others. Eventually, 116 farms were sampled. This is because some of the farms were not accessible after several attempts, other farmers refused to participate due to lack of feedback and benefits in earlier studies where they were involved. Of these farms, 65 (56%) were small-scale dairy farms while 51(44%) were poultry farms.

### Data collection

A structured questionnaire (Additional file 3) designed in English and digitalized into AfyaData, a mobile digital data application [38] was installed on a smartphone, pre-tested to minimise variations and improve its consistency to responses [39]. At the time of administration, it was translated in Kiswahili to farmers who did not know English in person on their farms? It included both closed & open-ended questions. The questionnaire consisted of three main sections: i) farmers socio-demographic characteristics ii) Cattle and iii) Poultry sections; farm attendants/owners were expected to give detailed information on numerous antimicrobial agents used in the past 5 months (in conjunction with the farmers’ treatment records and those of the livestock officer in charge) including: Route of application, purpose of use, drug withdrawal duration and vicinity of purchase. Other information obtained were product commercial name (which was classified by its active substance), livestock number, number of animals treated in the previous 5 months, type of antimicrobials used in the treatment doses, frequency of use, and number of days of treatment using recall or treatment records if at all they were available. The number of farms with antimicrobial use records and those which relied on recall use were noted. The obtained information was used to quantify the active Ingredients. To minimise recall bias, visual aid photos on the packages and labels for most antimicrobials used in livestock treatment were used.

### Data management and statistical analysis

Data from the questionnaires were downloaded into Microsoft Excel® 2007, cleaned, summarized and exported to IBM SPSS Statistics for windows, Version 2010 for further analysis. Descriptive statistics for all variables in the forms of frequencies and proportions were calculated. To determine the farmers level of antimicrobial use practices, each validated question was independently analysed and assigned a score. A desirable answer received a score of ‘one’ and undesirable ‘zero’ (Additional file 4).To analyse how each individual farmer Performed in the antimicrobial use practices, the sum of scores for each farmer was calculated which ranged between 0 and 12 where 0 is for a farmer who would score ‘0’ for each question and 12 for a farmer who would score ‘1’ for each question. From the sum of scores for each farmer a median score of seven (7) was computed and this was used as a cutoff point, such that any score < 7 was considered to be “Unfavorable” and ≥ 7 “Favorable”. Statistical associations were determined by Chi-square test and binary logistic regression analysis (BLRA) between categories whereby those with favorable practices constituted 58.6% and the rest unfavorable practices. The impact of farmers’ socio demographic profiles or association with respect to practices of antimicrobial agent usage was determined by binary logistic regression analysis. In order to be considered statistically significant, BLRA had to have a probability cut-off of 0.05. Odds ratios were calculated and then the 95% CI for the strength of association between variables was determined.

Antimicrobial use quantification was based on dose metrics. The quantity of active ingredient (milligrams) in each antimicrobial class were determined as follows: Active ingredients (mg) = no. of animals treated in the study period x antimicrobial drug concentration (mg/ml) x units per treatment (ml) x treatments per day x number of treatment days (for cattle) while for poultry was based on the quantity of drug consumed in water or feed multiplied by antimicrobial drug concentration (mg/ml). The daily water intake of 1 kg live chicken at ambient temperature of 320 C is 225 ml [40] while the daily feed consumption from Published data on a native Vietnamese layer is 63.4 g per kilogram of live chicken [41].Conversion factors for prodrugs (such as procaine benzyl penicillin) and international units (I.U.) were obtained from the ESVAC protocol [42]. The indiactors used were Defined Daily Dose (DDDvet mg/kg) and Used Daily Dose (UDDvet mg/kg) [43] which were calculated based on the farms’ antimicrobial use records or recall. A two group t-test was used to determine whether a significance difference existed between those who had records and those who relied on recall. The defined daily dose (DDD) as defined previously [44] as the average maintenance dose per day and per kg chicken/cattle of a specific drug, is determined on the basis of the instructions on drug’s leaflet. In case a single dose was indicated on the label, that was the DDDvet (mg/kg) and if a range of doses, the mean is the DDDvet. (mg/kg) [43]. For combined antimicrobials, the DDDvet value for each active ingredient was determined [45]. The standard weights considered for the study are those proposed by ESVAC [45].

The used daily dose (UDD), which defines a standard treated livestock/ animal [46] was determined by taking the average of the total amount of the administered antimicrobial compound in milligrams (mgs) and the number of animals times the mean weight at treatment. The ratio of UDD to DDD assesses the appropriateness of dosage as based on the investigation by Grave et al. [47]. Ratios within the range of 0.8 and 1.2 are considered as appropriate dosing while any value below 0.8 and above 1.2 are considered as under dosed and overdosed, respectively.

## Supplementary Information

**Additional file 1.**

**Additional file 2.**

**Additional file 3.**

**Additional file 4.**

## Data Availability

The datasets used and/or analysed during the current study are available from the corresponding author on reasonable request.

## Abbreviations

AMU: Antimicrobial use
AMR: Antimicrobial resistance
BLRA: Binary logistic regression analysis
CIs: Confidence Intervals
DDD: Defined Daily Dose
ESVAC: European Surveillance of Veterinary Antimicrobial Consumption
g: grams
I.U: International Unit
mg: milligrams
mg/kg: milligram/kilogram
mg/ml: milligram/milliliter
ml: milliliter
n: number
OR: Odds Ratio
*P*-value: Probability-value
%: Percentage
UDD: Used Daily Dose
