# Supplementary material for: Antimicrobial usage in cattle and poultry production in Dar es Salaam, Tanzania: pattern and quantity
Source: BMC Vet Res. 2022 Jan 3;18:7. doi: 10.1186/s12917-021-03056-9 (PMC8722348; doi:10.1186/s12917-021-03056-9)
Supplement: Supplementary file 2 — Additional file 2. [file 12917_2021_3056_MOESM2_ESM.docx]

**Additional file 2: Table 1** Antimicrobial drugs used among the 51 chicken farms surveyed in Dar es Salaam, Tanzania

| **Trade name** | **composition** |
| --- | --- |
| Agracox | Oxytetracycline 100 mg/g, Pyrimethamine 25 mg/g, Sulfadiazine sodium 25 mg/g , Sulfadimerazine sodium 200mg/g, and Vitamins A: 15.000 IU; K3: 5mg |
| Anticox | Sulfadimidine (sodium form) 74g and Diaveridine 8g |
| Coridix | Sulfamethoxypyridazine 125 mg/g, Trimethoprim 25 mg/g, Tylosin 30 mg/g, Sodium sulphate |
| Colistin | Colistin sulphate 4800000 IU Excipients ad 1g |
| Ciprofloxacin | Ciprofloxacin 200 mg. Excipients ad 1 ml |
| Doxycycline | Doxycycline (as hyclate) 100 mg Excipients ad 1 ml |
| Enrofloxacin | Each ml contains 100mg Enrofloxacin |
| Fluban | Enrofloxacin 100mg  Excipient q s 1ml |
| Flumequine | Each 100 gr contains : Flumequine 50 gr |
| Fluquin | Flumequine 200 mg, solvents 1 ml |
| Ganadexil 10% | Enrofloxacin, 100 mg; Excipient q.s. 1 ml. |
| Keproceryl wsp | Colistin (sulfate): 225000 IU, Oxytetracycline HCl: 5000 mg  Erythromycin thiocyanate: 35 mg Streptomycin sulfate: 35 mg and Vitamins: A,D3,E,K3,B1,B2 |
| Oxytetracycline | Oxytetracycline 50% |
|  |  |
| Trimazin | Trimethoprim 50 mg – Sodium sulfadiazine eq. 250 mg; Excipients up to 1 g |
| Tylo-dox Extra wsp | Contains per gram powder Doxycycline hyclate 200 mg. Tylosin tartrate 100 mg. Excipients ad 1 g |
| Tylosin | Tylosin tartrate 200 mg/g |
| Typhoprim | Sodium sulfadiazine,250 mg/g; Trimethoprim 50 mg/g |

**Table 2** Type of antimicrobials used in the 51 chicken farms surveyed in Dar es Salaam, Tanzania

| Class of antimicrobial | Antimicrobial name | Number of drugs (%) administered containing antimicrobials | Number of farms (%) using the antimicrobial |
| --- | --- | --- | --- |
| Aminoglycosides | Streptomycin | 1 (5.9) | 1 (2.0) |
| Diaminopyrimidines | Trimethoprim | 3 (17.6) | 9 (17.6) |
| Fluoroquinolones | Ciprofloxacin | 1 (5.9) | 19 (37.3) |
|  | Enrofloxacin | 3 (17.6) |  |
|  | Flumequine | 2 (11.8) |  |
| Macrolides | Erythromycin | 1 (5.9) | 10 (19.6) |
|  | Tylosin | 2(11.8) |  |
| Polymyxins | Colistin | 2 (11.8) | 2 ( 3.9) |
| Sulphonamides | Sulfadiazine | 3 (17.6) | 12 ( 23.5) |
|  | Sulfadimidine | 1 (5.9) |  |
|  | Sulfadimerazine | 1 (5.9) |  |
|  | Sulfamethoxypyridazine | 1 (5.9) |  |
| Tetracyclines | Doxycycline | 2 (11.8) | 13 (24.5) |
|  | Oxytetracycline | 4 (23.5) |  |

**Table 3** Antimicrobial drugs used among 65 small scale dairy farms surveyed in Dar es Salaam, Tanzania

| **Antimicrobial class** | **Antimicrobial name** | **composition** |
| --- | --- | --- |
| Beta-lactamase |  |  |
|  | Penicillin | Procaine Benzyl penicillin 300mg/ml |
|  |  |  |
|  | Ampicillin | Ampicillin trihydrate 500mg/ml |
| Aminoglycosides |  |  |
|  | Gentamicin | Gentamicin sulphate 100000 I.U. Excipients up to 1 ml. |
|  | Neomycin | Each mL contains 200 mg of neomycin sulfate equivalent to 140 mg neomycin base. |
|  | Penstrep | Dihydrostreptomycin Sulphate 250mg/ml  Procaine Penicillin 200mg/ml |
| Fluoroquinolones |  |  |
|  | Enrofloxacin | Baytril 10% Injectable  Solution Enrofloxacin 100mg/ml |
| Macrolides |  |  |
|  | Tylosin | Tylosin Tartrate 200mg/ml |
| Sulphonamides |  |  |
|  | Ashulpha | Sulfadimidine 333mg/ml |
|  | Co-Trimoxazole | Sulfamethoxazole 200mg/ml |
|  |  | Trimethoprim 40mg/ml |
|  | Intertrim | Trimethoprim 40 mg/ml  Sulfamethoxazole 200 mg/ml |
|  | Kombitrim | Sulfamethoxazole 200 mg/ml  Trimethoprim 40 mg/ml |
|  | Norodine | Trimethoprim 40 mg/ml  Sulfadiazine 200mg/ml |
| Tetracyclines |  |  |
|  | Oxytetracycline | Oxytetracyclline Hydrochloride 100 mg/ml |

**Table 4** Type of antimicrobials used in the 65 small scale dairy farms surveyed in Dar es Salaam, Tanzania

| Class of Antimicrobial | Name of antimicrobial | Number of drugs (%) administered containing antimicrobials | Number of farms (%) using the antimicrobial |
| --- | --- | --- | --- |
| Beta-lactam | Procaine Benzyl penicillin | 1 (7.7) | 12 ( 18.5) |
|  | Ampicillin | 1 (7.7) | 3 (4.6) |
|  |  |  |  |
| Aminoglycosides |  |  |  |
|  | Gentamicin | 1 (7.7) | 7 (10.8) |
|  | Neomycin | 1 (7.7) | 2 (3.1) |
|  | Penstrep | 1 (7.7) | 7 (10.8) |
| Diaminopyrimidines |  |  |  |
|  | Trimethoprim | 3 (23.1) | 6 (9.2) |
|  |  |  |  |
| Fluoroquinolones |  |  |  |
|  | Enrofloxacin | 1 (7.7) | 5 (7.7) |
|  |  |  |  |
| Macrolides |  |  |  |
|  | Tylosin | 1 (7.7) | 8 (12.3) |
|  |  |  |  |
| Sulphonamides |  |  |  |
|  | Sulfadiazine | 1 (7.7) | 1 (1.5) |
|  | Sulfadimidine | 1 (7.7) | 1 (1.5) |
|  | Sulfamethoxazole | 3 (23.1) | 6 (9.2) |
|  |  |  |  |
| Tetracycline |  |  |  |
|  | Oxytetracycline | 1 (7.7) | 13 (20.0) |
