# Supplementary material for: Antimicrobial usage in cattle and poultry production in Dar es Salaam, Tanzania: pattern and quantity
Source: BMC Vet Res. 2022 Jan 3;18:7. doi: 10.1186/s12917-021-03056-9 (PMC8722348; doi:10.1186/s12917-021-03056-9)
Supplement: Supplementary file 3 — Additional file 3. [file 12917_2021_3056_MOESM3_ESM.docx]

**Additional file 3**

**QUESTIONNAIRE**

This questionnaire is meant to assess the level of practice of antimicrobial usage among cattle and poultry farmers in Dar-es-Salaam, Tanzania. Participation is voluntary and all information given will be kept strictly confidential. *PLEASE* ***tick one or more*** *appropriately and* ***write*** *where necessary****.***

**IDENTIFICATION**

Date:________________________ Name of interviewer:_______________________________

Region:_______________________ District:___________________ Ward:________________

**FARM (HOUSEHOLD) CHARACTERISTICS/ DEMOGRAPHIC INFORMATION**

1. Name of interviewee: ____________________________

2. Age (in years):_____________

3. Gender: a. Male ( ) b. Female ( )

4. Level of education:

a. Informal ( ) b. Primary ( ) c. Secondary ( ) d. Tertiary ( )

5. Main Occupation of respondent: a. Livestock ( ) b. Others ( )

6. Experience in livestock rearing/ management.

1. Less than six years ( )
2. More than six years ( )

7. Type of domestic animal/poultry do you keep

a. Cattle ( )

b. Chicken ( )

c. Cattle & chicken ( )

**CATTLE SECTION**

1. **Practices regarding Antimicrobial usage in Dairy Cattle**

A1. Have you used antimicrobials on your dairy farm? a. ­­­­ Yes ( ) b. No ( )

A2. Do you get them over the counter? a. Yes ( ) b. No ( )

b. If yes who prescribes them for you? a. Self ( ) b. Neighbour ( ) c. Veterinarian

A3. Which of the following antibiotics have ever been used at this farm?

| **Antimicrobial** | **Tick** |  | **Antimicrobial** | **Tick** |
| --- | --- | --- | --- | --- |
| Tetracycline (any) |  |  | Ampicillin |  |
| Penicillin |  |  | Enrofloxacin |  |
| Gentamicin |  |  | Chloramphenicol |  |
| Neomycin |  |  | Penstrep |  |
| Ciprofloxacin |  |  | Unknown |  |
| Sulfur based (any) |  |  | Other specify |  |

A4. Which antibiotics among the mentioned above do you use most and why________________

A5. Who treats your cattle once they fall sick?

a. Self ( ) b. Neighbour ( ) c. Veterinarian ( )

A6. What amounts were administered in Q.A4 above?__________________

A8. Where do you normally buy veterinary antibiotics?

a. Veterinary drug shop ( ) b. Veterinary clinic ( ) c. Individual veterinarian ( )

A8. Do drug sellers usually ask for prescription when selling veterinary antibiotics?

a. Yes ( ) b. No ( ) c. Sometimes

A9. Do you keep antimicrobials on your farm? a. Yes ( ) b. No ( )

A10. If yes, where do you store them?

a. Cupboard ( ) b. Open shelf in doors ( ) c. Shelf direct sunlight ( )

A11. Do you usually comply with antimicrobial drug withdraw periods?

a. Yes ( ) b. No ( )

A12. What is the purpose of antimicrobial use?

a. Treatment ( ) b. Prevention ( ) c. Treatment & Prevention ( ) d. Growth Promotion ( )

**POULTRY SECTION**

**B. Practices regarding Antimicrobial usage**

B1. Have you used antimicrobials on your poultry farm? a. ­­­­ Yes ( ) b. No ( )

B2. Do you get them over the counter? a. Yes ( ) b. No ( )

Bb. If yes who prescribes them for you?

a. Self ( ) b. Neighbour ( ) c. Veterinarian ( )

B3. Please specify the common antibiotics you use?

| **Antimicrobial** | **Tick** |
| --- | --- |
| Tetracycline |  |
| Penicillin |  |
| Amoxicillin |  |
| Streptomycin |  |
| Gentamicin |  |
| Ciprofloxacin |  |
| Enrofloxacin |  |
| Tylosin |  |
| Polymyxins |  |
| Trimethoprim |  |
| Others specify |  |

B4. Where do you usually buy the antimicrobials from?

a. Veterinary drug shops ( ) b. Veterinary clinic ( ) c. Individual veterinarian ( )

B5. Do drug sellers ask for prescriptions?

a. Yes ( ) b. No ( ) c. Sometimes ( )

B6. Who administers antimicrobials used on poultry in your flock?

a. Self ( ) b. Neighbours ( ) c. ( ) Veterinarian

B7. For what purpose are antimicrobials used for?

a. Treatment ( ) b. Prevention ( ) c. Treatment & Prevention ( ) d. Growth Promotion ( )

B8. What common route of antimicrobial administration do you use?

a. Parenteral route ( ) b. Orally ( ) c. Feed ( )

B9. Do you Keep records? a. Yes ( ) b. No ( )

Bb. If yes, which one? a. Treatment ( ) b. Purchase ( )

B10. Do you record the volumes or quantities of antimicrobial used? a. Yes ( ) b. No ( )

B11. Do you observe the withdrawal period?

a. Yes ( ) b. No

B12. Do you store antimicrobials on your farm? a. Yes ( ) b. No ( )

b. If yes how? A. Cupboard ( ) b. Shelf in doors ( ) c. Shelf direct sunlight

B13. Do drug sellers usually ask for Prescriptions? a. Yes ( ) b. No ( ) c. Sometimes

Thank you for the kind responses

**For further information, contact:**

**E-mail:** [rogersazabo@gmail.com](mailto:rogersazabo@gmail.com); **Mobile Tel no:** +255 654 641 180
