# Supplementary material for: Antimicrobial usage in cattle and poultry production in Dar es Salaam, Tanzania: pattern and quantity
Source: BMC Vet Res. 2022 Jan 3;18:7. doi: 10.1186/s12917-021-03056-9 (PMC8722348; doi:10.1186/s12917-021-03056-9)
Supplement: Supplementary file 4 — Additional file 4. [file 12917_2021_3056_MOESM4_ESM.docx]

**Additional file 4**: A questionnaire to small scale dairy cattle and poultry (chicken) keepers on Antimicrobial (Antibiotic) Use and related practices in Dar es Salaam, Tanzania

Date_______________________________Interviewer________________________________

District__________________Ward_______________Interviewee_______________Sex______

| No | Questions | Levels | Responses |
| --- | --- | --- | --- |
| 1 | Do you consume milk/eggs from dairy cattle/  Poultry that were just treated with  antimicrobials? | Desirable (1)  Undesirable (0) | No  Yes |
| 2 | Do you consume meat from dairy cattle /poultry  that was just treated with antimicrobials? | Desirable (1)  Undesirable (0) | No |
|  |  |  | Yes |
| 3 | How long do use antimicrobials in dairy cattle/  /poultry? | Desirable (1) | As advised |
|  |  | Undesirable (0) | Until cattle/poultry is cured; Until the bottle/vial/package is empty; As long as I can afford; Once(one) time treatment; Continuously over extended period |
| 4 | What do you do with expired veterinary antimicrobials? | Desirable (1) | Dispose of; Return to the veterinary drug shop/pharmacy; Refuse to receive it |
|  |  | Undesirable (0) | Give it to other farmers; Use it for intended treatment; Nothing |
| 5 | How do you use manure? | Desirable (1) | Used as fertilizer; used as fuel (biogas); Sold for cash (fuel) |
|  |  | Undesirable (0) | Leave on farm in open air; discard into the environment |
| 6 | Do you have isolation unit for sick animals/birds? | Desirable (1)  Undesirable (0) | Yes  No |
| 7 | Do veterinary drug sellers ask for prescriptions? | Desirable (1)  Undesirable (0) | No  Yes,sometimes |
| 8 | What do you do when cattle/poultry dies on the farm? | Desirable (1) | Bury, burn |
|  |  | Undesirable (0) | Leave as it is; give to  the dog; home  consumption |
| 9 | Who provides source of drug information to the farmer? | Desirable (1) | Veterinarian; Livestock health practitioner |
|  |  | Undesirable (0) | Myself; neighbour; fellow farmer |
| 10 | Where do you purchase antimicrobials? | Desirable (1) | Veterinary drug shops; Agro-vet shops; Veterinary clinics, Individual veterinarians |
|  |  | Undesirable (0) | Livestock market vendors; informal drug sellers/dealers |
| 11 | Who administers the antimicrobials? | Desirable (1) | Veterinarian; Livestock health practitioner |
|  |  | Undesirable (0) | Myself, neighbour |
| 12 | Where do you store antimicrobials bought/brought on the farm? | Desirable (1) | Cupboard (cool & dry place) |
|  |  | Undesirable (0) | Open shelf indoor;  Shelf direct sunlight |
